# Cardamonin inhibits the progression of oesophageal cancer by inhibiting the PI3K/AKT signalling pathway

**DOI:** 10.7150/jca.55519

**Published:** 2021-04-24

**Authors:** Zijie Wang, Hui Liu, Qing Hu, Lei Shi, Muhan Lü, Mingming Deng, Gang Luo

**Affiliations:** 1 From the Department of Gastroenterology, Affiliated Hospital of Southwest Medical University, Luzhou, Sichuan 646000, China.; 2 Nuclear Medicine and Molecular Imaging Key Laboratory of Sichuan Province, Luzhou, Sichuan, 646000, China.

**Keywords:** oesophageal cancer, cardamonin, PI3K/AKT signalling pathway, antitumour, growth

## Abstract

**Background:** Oesophageal cancer is the most common malignant tumour with a poor prognosis, and the current treatment methods are limited. Therefore, identifying effective treatment methods has become a research hotspot. Cardamonin (CAR) is a natural chalcone compound and has been reported to play an anticancer role in several cancers. However, its function in oesophageal cancer and the possible underlying mechanism are still unclear. The purpose of this study was to demonstrate the anticancer effect of CAR on oesophageal cancer *in vivo* and *in vitro* and to explore the underlying mechanism.

**Materials and Methods:** MTT, crystal violet, and colony formation assays were used to detect oesophageal cancer cell proliferation. The effects of CAR on oesophageal cancer cell migration and invasion were detected by wound healing assay and Transwell assay. Hoechst 33258 staining and flow cytometry were used to detect cell apoptosis. Protein expression levels were detected by Western blot. A tumour xenograft model was established to further test the effect of CAR on the growth of oesophageal cancer *in vivo*.

**Results:** The results showed that CAR inhibited the proliferation, migration, and invasion of oesophageal cancer cells in a concentration-dependent manner and induced apoptosis. Furthermore, the Western blot assay showed that CAR could suppress metastasis by inhibiting epithelial-mesenchymal transition (EMT) as indicated by downregulated expression of the mesenchymal markers N-cadherin and vimentin, the EMT transcription factor Snail, and matrix metalloproteinases (MMPs) and upregulated expression of the epithelial marker E-cadherin. CAR was associated with upregulation of the pro-apoptotic proteins Bax and Bad and downregulation of the anti-apoptotic protein Bcl-2 and triggered the mitochondrial apoptosis pathway, which in turn promoted caspase-3 activation and subsequent cleavage of PARP; however, the mitochondria-related apoptotic effects induced by CAR were blocked by caspase inhibitor Z-VAD-FMK pretreatment, which prevented programmed cell death triggered by CAR. In addition, CAR reduced the phosphorylation level of downstream effector molecules of phosphatidylinositol 3 kinase (PI3K) in a dose-dependent manner, and treatment with the PI3K agonist 740Y-P could partially reverse the anticancer effect of CAR, demonstrating that CAR played an antitumour role by inhibiting the PI3K/AKT signalling pathway in oesophageal cancer cells. Moreover, the EC9706 xenograft model further confirmed that CAR can significantly inhibit tumour growth *in vivo*.

**Conclusion:** In summary, CAR exhibited a strong anticancer effect on human oesophageal cancer cells and promoted apoptosis by inhibiting the PI3K/AKT signalling pathway, suggesting that CAR can be used as new strategy for oesophageal cancer treatment.

## Introduction

Oesophageal cancer is one of the most common malignant tumours in the world and is considered the most invasive gastrointestinal malignant tumour in the world. Its incidence ranks seventh among malignant tumours worldwide, and the mortality rate ranks sixth 1. Oesophageal cancer includes two main histological subtypes: oesophageal squamous cell carcinoma and oesophageal adenocarcinoma, with the former being the dominant subtype worldwide 2. The early symptoms of oesophageal cancer are mostly atypical. Patients have already reached the middle and advanced stages when symptoms such as progressive dysphagia and swallowing pain appear. Although surgery is still the main method for the treatment of oesophageal cancer, it is very invasive 3. At the middle and advanced stages, comprehensive treatments such as surgery, biological agents, immunization, chemotherapy, and radiotherapy are typically adopted. However, due to serious postoperative adverse reactions, chemotherapy resistance, and disease recurrence, these treatment methods are unsatisfactory 4. Therefore, the search for effective drug treatments has become a research hotspot. Substances extracted from natural plants have been regarded as potential antitumour drugs due to their strong antitumour effects and few side effects 5-6.

In the past few decades, a total of 155 anticancer drugs have been approved for clinical use by the US Food and Drug Administration (FDA), 14% of which are derived from natural products, while 28% are semi-synthetic analogues of natural products, include vinca alkaloids, vincristine, vinblastine, paclitaxel, and their derivative compounds 7-8. Cardamonin (CAR) is a small molecule chalcone compound extracted from plants belonging to the genus *Alpinia* of the family Zingiberaceae, such as cardamom 9. Numerous studies have shown that CAR has a variety of biological activities, including anti-inflammatory, antioxidant, antitumour, and anti-infection effects, blood vessel dilation effects, hypoglycaemic effects, and lipogenesis inhibition effects 9-11. CAR has been reported to have low toxicity to normal cells and to exert different degrees of antitumour effects on tumour cells. Therefore, CAR is considered a promising novel drug candidate for cancer treatment 12. CAR has also been shown to have antitumour activity through the regulation of a variety of cell signalling pathways 13. CAR not only suppressed HIF-1α-mediated cell metabolism by inhibiting the mTOR/p70S6K signalling pathway but also induced the expression of target genes, including p21, p27, bim, and activated caspase-3, and led to a reduction in cyclin D1 through the JNK-FOXO3a pathway, thus inhibiting the growth of breast cancer cell line MDAMB-231 *in vivo* and *in vitro* 14-15. CAR, as a potential STAT3 blocker, has been reported to inhibit STATS phosphorylation, nuclear translocation, and DNA binding and to bind directly to the Src Homology 2 (SH2) domain of STAT3 and effectively inhibit its dimerization, significantly mitigating the growth and survival of prostate cancer cells and glioblastoma stem cells 16,22. CAR was reported to inhibit HCT116 cell proliferation, induce cell cycle arrest in the G2/M phase, and enhance autophagy by activating c-Jun N-terminal kinase (JNK) 17. CAR inhibited the proliferation and metastasis of non-small cell lung cancer A549 and H460 cells by reducing the phosphorylation level of the downstream effector phosphoinositide 3 kinase (PI3K) 19. The effects of CAR on nasopharyngeal carcinoma cells are related to apoptosis and G2/M phase arrest through inhibition of NF-κB pathway activation, which in turn triggers reactive oxygen species (ROS) accumulation to activate JNK mitogen-activated protein kinase (MAPK) 20. The anticancer effects of CAR on cancer cells are related to apoptosis induction, as well as inhibition of proliferation, migration, and cell cycle progression. CAR can also reduce the chemotherapy resistance of cancer cells. CAR combined with 5-fluorouracil and cisplatin can enhance the antitumour activity of chemotherapy drugs. For example, CAR can significantly inhibit the growth of chemotherapy-resistant colon cancer cells, induce apoptosis, promote caspase-3/9 activity and Bax protein expression, and significantly inhibit the expression of c-myc, testis-specific protease 50, and nuclear factor-κB protein 18. CAR combined with cisplatin can enhance antiproliferation efficacy by suppressing mTOR activation in SKOV3 and A2780 cells 21. CAR can result in the apoptosis and cell cycle arrest of BGC-823/5-FU cells by reducing the expression of P-glycoprotein and β-catenin protein and interrupting β-catenin/TCF4 complex formation 23. Because of the obvious antitumour effects of CAR, it has received extensive attention in recent years. However, the roles and specific mechanism of CAR in oesophageal cancer have not been systematically investigated.

The PI3K/AKT signalling pathway is considered one of the most critical intracellular signalling pathways. This pathway plays important roles in the physiological and pathological processes of cells and is involved in tumourigenesis, proliferation, apoptosis, metabolism, metastasis, angiogenesis, and drug resistance 24. Studies have shown that PI3K/AKT/mTOR is highly activated in oesophageal cancer cells 25. CAR has been reported to inhibit the proliferation and metastasis of non-small cell lung cancer cells through inhibition of the PI3K/AKT/mTOR pathway 19. However, no studies have reported whether CAR inhibits oesophageal cancer by regulating the PI3K/AKT pathway.

In this study, we confirmed the antitumour effect of CAR on oesophageal cancer cells for the first time and explored the possible molecular mechanism. Our results indicated that CAR achieved anti-oesophageal cancer cell effects by inhibiting abnormal activation of the PI3K/AKT signalling pathway.

## Materials and methods

### Reagents and materials

EC-9706 and TE10 human oesophageal cancer cells were gifted by the Medical Experimental Centre of Southwest Medical University, China. CAR (purity > 98%, Chengdu Herbpurify Co., Ltd., China) was dissolved in dimethyl sulfoxide (DMSO; Shanghai Beyotime Biotechnology Co., Shanghai, China) and stored at -80 °C. RPMI-1640 medium, foetal bovine serum (FBS), penicillin, and streptomycin were provided by Gibco Life Technologies (Grand Island, NY, USA), and 3-(4,5-dimethylthiazol-2-yl)-2,5-diphenyltetrazolium bromide (MTT) was purchased from Sigma-Aldrich (St. Louis, Missouri, USA). Crystal violet staining solution was purchased from Solarbio Life Sciences (Beijing, China). Mouse anti-human β-actin antibody, Hoechst 33258 staining solution, trypsin solution (0.25% trypsin), and bicinchoninic acid (BCA) protein assay kits were all purchased from Shanghai Beyotime Biotechnology (Shanghai, China). Annexin V-FITC/propidium iodide (PI) apoptosis assay kits and Matrigel were purchased from Biosciences (USA). Transwell chambers were purchased from Corning Incorporated (USA). Z-VAD-FMK and 740Y-P were purchased from Dalian Meilun Biotech Co., Ltd. (Dalian, China). Balb/c-nude female mice were purchased from Chengdu Dashuo Biotechnology Co., Ltd. All animal experiments were approved by the Ethics Committee of Southwest Medical University. For Western blot analysis, rabbit anti-human PARP, cleaved PARP, caspase-3, cleaved caspase-3, N-cadherin, E-cadherin, MMP2, MMP9, MMP7, PI3K, p-PI3K, AKT, p-AKT, vimentin, and PCNA, other monoclonal antibodies, and mouse anti-human monoclonal antibodies such as Bcl2, Bax, vimentin, and Snail were all purchased from Abcam (Cambridge, UK), Horseradish peroxidase (HRP)-labelled goat anti-rabbit and goat anti-mouse IgG (secondary antibody) were purchased from Boster (Wuhan, China).

### Cell culture and treatment

Oesophageal cancer cells were cultured in RPMI-1640 medium containing 1% bispecific antibody and 10% FBS. The cells were cultured in an incubator at 37 °C and in 5% CO_2_, and cells in the logarithmic growth phase were selected for subsequent experiments. The experimental control group (DMSO group) was treated with DMSO only, with a final concentration ≤ 0.1%, similar to that in the experimental groups, while the negative control group (NC group) was not treated. Different concentrations of CAR were used to treat cells for 24 h and 48 h.

### Crystal violet staining

Oesophageal cancer cells at a density of 3×10^4^ cells/well were seeded in 24-well plates. Then, the cells were treated with different concentrations of prepared CAR or DMSO, and the NC group was set up. After incubation in an incubator for 24 h and 48 h, crystal violet staining solution was added to each well. After imaging under a scanner, the cells were fully dissolved in 20% acetic acid solution. To calculate cell viability, the optical density (OD) value of each well was measured at 590 nm using an enzyme-linked immunosorbent assay (ELISA) spectrophotometer.

### MTT assay

EC9706 and TE10 oesophageal cancer cells in the logarithmic growth phase were harvested and inoculated into 96-well plates at a density of 3000 cells/well. The cells were cultured in an incubator for 12 h and then treated with different concentrations of CAR and DMSO. The NC group was also set up. After incubation for 24 h or 48 h, 10 µL of MTT solution (5 mg/ml) was added to each well. The supernatant was aspirated, and 100 µL of DMSO was added to each well. The absorbance at a wavelength of 490 nm was measured using a microplate reader.

### Colony formation assay

Oesophageal cancer cells were inoculated into 6-well plates at a density of 8×10^2^ cells/well and treated with different concentrations of CAR. The cells were continuously cultured for two weeks until colonies visible to the naked eye had formed. Then, the supernatant was discarded, and crystal violet staining solution was added for 10 min. The cells were dried and photographed, and the colony formation rate was calculated.

### Flow cytometry

After the oesophageal cancer cells were treated with different concentrations of CAR for 48 h, the cells were digested with trypsin, washed twice with PBS, and resuspended in 1× loading buffer. The cells were double-stained with 5 µL of annexin V-FITC and 5 μL of PI and then incubated in the dark for 30 min before flow cytometry analysis.

### Hoechst 33258 staining

Cells were seeded into 24-well plates (3×10^4^ cells/well). The cells were treated with different concentrations of CAR and then incubated for 48 h, followed by fixation with 4% paraformaldehyde for 30 min and one wash with PBS. The cells were stained with Hoechst 33258 (50 ng/ml) for 30 min. Apoptotic cells were observed using a fluorescence microscope (apoptotic cells were characterized by dense chromatin), and five randomly selected fields were used for cell counting.

### Wound healing assay

Oesophageal cancer cells were seeded into 6-well plates (5×10^5^ cells/well) and then linearly scratched with a 10-μL pipette tip. After treatment for 24 h with different concentrations of CAR, cell migration was observed using an inverted microscope.

### Transwell migration and invasion assays

A total of 100 μL of Matrigel was placed in the upper chamber of a Transwell system and incubated at 37 °C for 2 h to form a white membrane visible to the naked eye. Cells were resuspended in serum-free medium and adjusted to a concentration of 3×10^4^ cells/well. Medium containing 10% FBS was added into the lower chamber, and CAR was added based on a concentration gradient. Next, the cells were incubated for 48 h and then stained with crystal violet for 10 min. Then, an inverted microscope was used to observe the cells; five fields were randomly selected to observe and count the cells. To assess cell migration, all the above steps were repeated, but Matrigel was not added to the upper chamber.

### Western blot analysis

Oesophageal cancer cells were inoculated into a 6-well plate (5×10^5^ cells/well). Cells were treated with different concentrations of CAR and then incubated for an additional 48 h. The cells were lysed using radio-immunoprecipitation assay (RIPA) buffer. The protein concentration was determined using a BCA kit. Proteins were separated by 8-12% sodium dodecyl sulfate-polyacrylamide gel electrophoresis (SDS-PAGE) and then transferred to a polyvinylidene fluoride (PVDF) membrane. The membrane was blocked with 5% nonfat milk for 1 h and then incubated with primary antibodies at 4 °C overnight. The membrane was then incubated with secondary antibodies for 1 h at room temperature. Finally, the membrane was imaged using a chemiluminescence imaging analyser. The greyscale value of each target protein band was analysed using the software provided with the analyser.

### Animal model

A suspension of EC9706 cells (2×10^7^ cells/ml) was injected into the middle of and posterior to the armpit of 4- to 6-week-old male nude mice. One week after inoculation, a soybean-sized subcutaneous mass appeared in the armpits of the mice. Then, different doses of CAR (5, 15, or 25 mg/kg) or sodium carboxymethyl cellulose (CMC), which served as a control, were administered by gavage once every three days. The body weights of the mice and tumour size were measured at the same time. Mice were euthanized 26 days after drug administration. Immunohistochemistry (IHC) was used to stain the tumours, and stomach tissue was stained with haematoxylin-eosin (HE).

### Immunohistochemistry (IHC) and haematoxylin-eosin (HE) staining

Tumour tissues were fixed with 4% paraformaldehyde and embedded in paraffin. Paraffin-embedded tissue blocks were cut into slices with a thickness of 3-5 mm. Tumour sections were immunohistochemically stained with PCNA (1:100), Bcl-2 (1:100), vimentin (1:100), phosphorylated Akt (1:100), and phosphorylated PI3K (1:100) antibodies according to the steps of the immunohistochemistry kit. For HE staining, tissues were sectioned after deparaffinization, stained with haematoxylin and eosin, and mounted on slides with neutral gum.

### Statistical analysis

All data are expressed as the mean ± standard deviation (SD) of at least three independent experiments. Differences among groups were analysed by one-way analysis of variance (ANOVA) using SPSS 24.0 software (SPSS, Chicago, IL, USA). Differences with p < 0.05 were considered statistically significant.

## Results

### CAR inhibited oesophageal cancer cell proliferation

**Figure 1:**
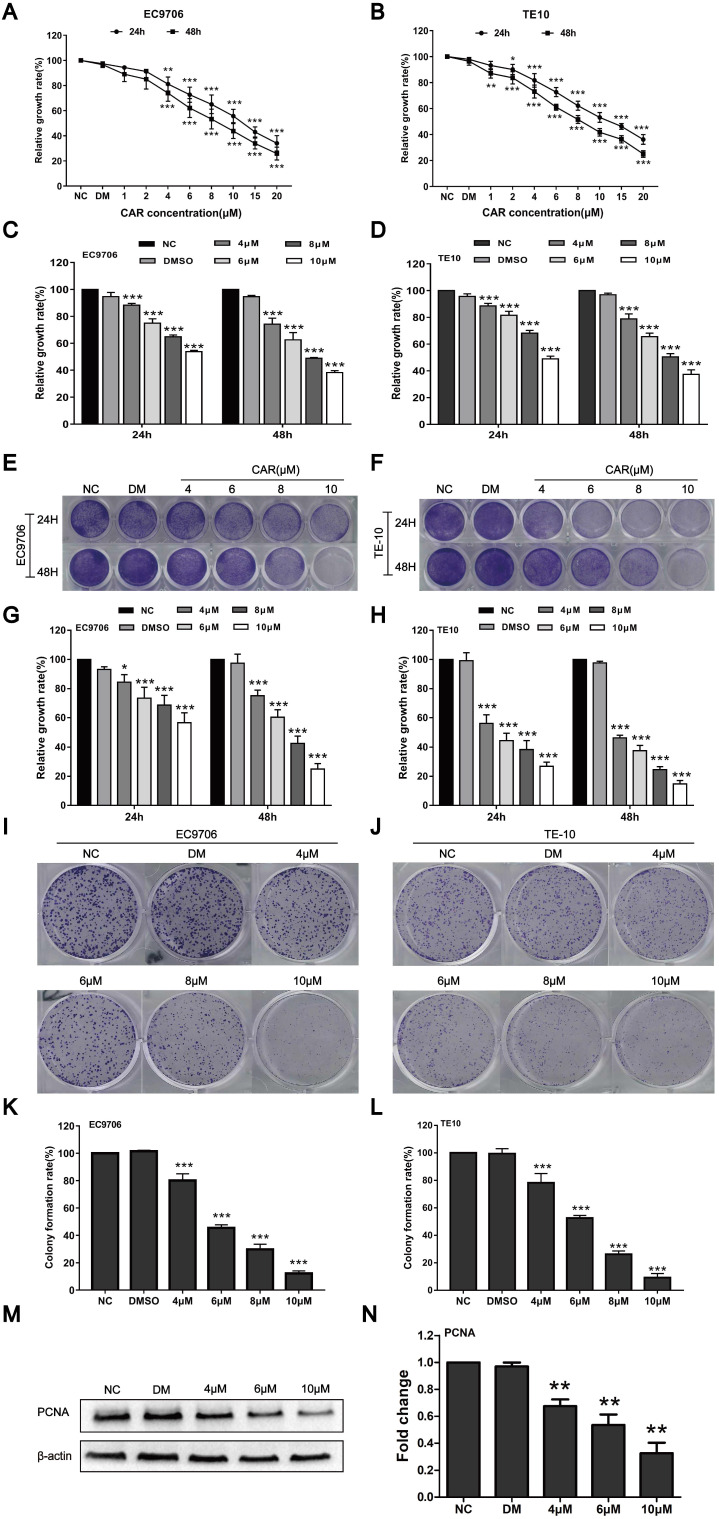
CAR inhibited oesophageal cancer cell proliferation. (A and B) EC9706 and TE10 cells were treated with various dosages of CAR, and the cell growth rate was examined by MTT assay; with increases in the drug concentration and treatment time, the growth of EC9706 and TE10 cells was obviously inhibited. The cells were treated with the screened CAR concentration, and (C and D) MTT assay and (E-H) crystal violet staining results further verified that CAR inhibited the growth of oesophageal cancer cell lines EC9706 and TE10 in concentration- and time-dependent manners. (I-L) Colony formation assay showed that CAR inhibited the growth of oesophageal cancer cell lines EC9706 and TE10 in a concentration-dependent manner. (M and N) Western blot assay results showed that PCNA protein expression in EC9706 cells decreased with increasing CAR concentrations with β-actin as the loading control. All the above quantitative results are presented as the mean ± SD (n = 3, each group). *p < 0.05, **p < 0.01, ***p < 0.001 vs the NC group.

To study the effect of CAR on oesophageal cancer cell proliferation, we first detected the survival rates of EC9706 and TE10 oesophageal cancer cells after treatment with different drug concentrations and DMSO by MTT assay. Compared with the DMSO and NC groups, the CAR group had a markedly decreased survival rate. The MTT method was used to screen drug concentrations (Fig. 1A and B) and determine the half maximum inhibitory concentration (IC50) of CAR. As shown in (Supplementary Fig. 1A-D), the IC50 values of EC9706 cells at 24 h and 48 h were 12.25 µM and 8.674 µM, respectively, while the IC50 values of TE10 cells at 24 h and 48 h were 12.48 µM and 8.405 µM, respectively. To investigate the effect of CAR on normal cells, we measured the IC50 values of CAR in human normal oesophageal cells (HET-1A) and normal gastric epithelial cells (GES-1). We found that CAR had higher IC50 values in these two cell lines, which may suggest that CAR had little effect on normal cells (Supplementary Fig. 1E-H). Based on the above data, we selected 4, 6, 8, and 10 µM as the working concentrations for subsequent experiments with EC9706 and TE10 cells. The antiproliferative effect of CAR was confirmed by crystal violet staining, as shown in (Fig. 1E-H), similar to the MTT assay (Fig. 1C and D), CAR inhibited oesophageal cancer cell proliferation in time- and concentration-dependent manners. In addition, we further validated the results using a colony formation assay. As shown in (Fig. 1I-L), CAR had a significant inhibitory effect on the colony formation of oesophageal cancer cells in a concentration-dependent manner, which was consistent with previously observed antiproliferative effects. Finally, Western blot analysis was used to detect the expression of PCNA, a marker of cell proliferation, in EC9706 cells. As shown in (Fig. 1M and N), CAR significantly inhibited PCNA expression. The above experimental results suggest that CAR inhibited oesophageal cancer cell proliferation.

### CAR induced oesophageal cancer cell apoptosis

**Figure 2:**
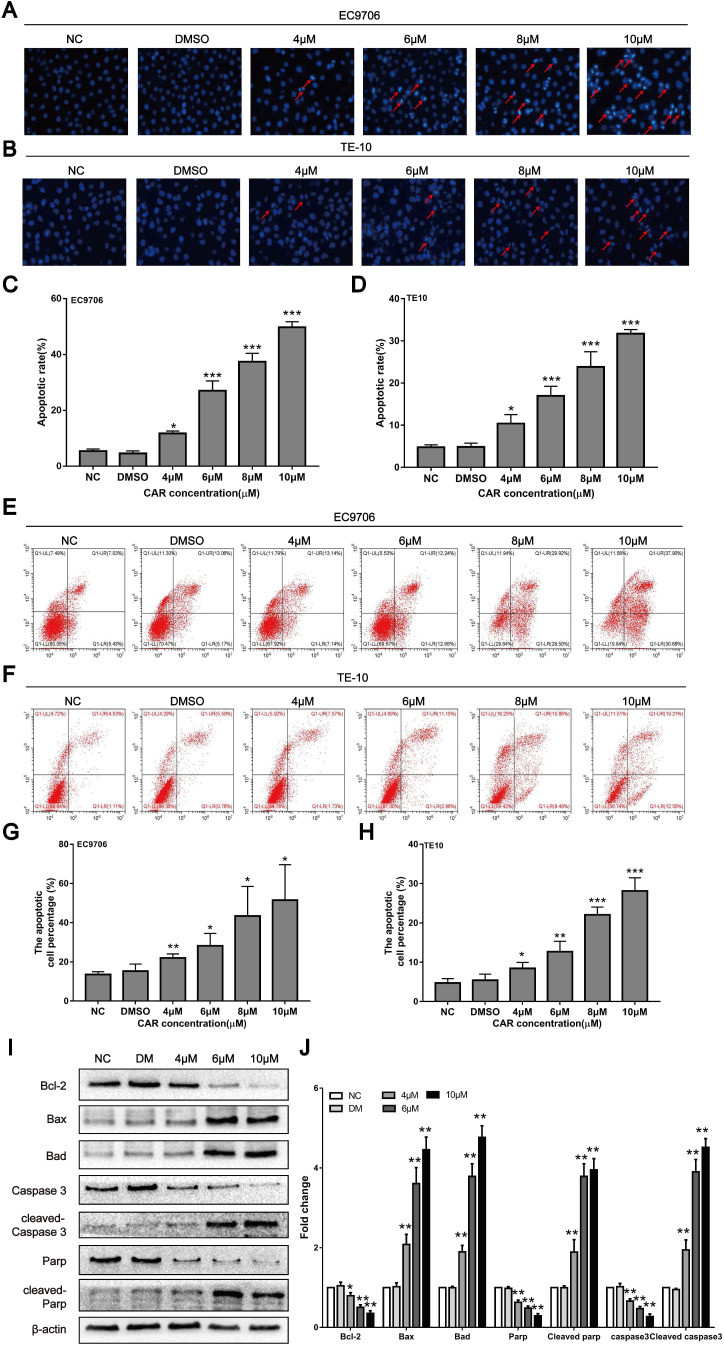
CAR induced oesophageal cancer cell apoptosis. (A-D) EC9706 and TE10 cells were treated with different concentrations of CAR or DMSO for 48 h and then stained with Hoechst 33258. Apoptotic cells with dense chromatin were statistically analysed, and the apoptosis rate was quantified. (E-H) Flow cytometry was used to detect the effect of CAR on oesophageal cancer cell apoptosis. The results proved that CAR induced apoptosis in a concentration-dependent manner. (I and J) The expression levels of apoptosis-related proteins were detected by Western blot with β-actin as the loading control, and with increasing CAR concentrations, the expression levels of cleaved caspase-3, cleaved PARP, Bax, and Bad were upregulated, while the expression levels of caspase-3, PARP, and Bcl2 were downregulated. All the above quantitative results are presented as the mean ± SD (n = 3, each group). *p < 0.05, **p < 0.01, ***p < 0.001 vs the NC group.

Previous studies have shown that CAR can exert antitumour activity by inducing apoptosis in cancer cells. Therefore, we first examined whether CAR inhibited oesophageal cancer cell apoptosis using Hoechst 33258 staining. Cells containing dense chromatin with dense staining were considered apoptotic. As shown in (Fig. 2A-D), compared with that in the control group, the number of apoptotic EC9706 and TE10 oesophageal cancer cells gradually increased with increasing drug concentrations. In addition, we used annexin V-FITC/PI double staining and flow cytometry to further validate the effect of CAR on oesophageal cancer cell apoptosis. The results showed that CAR increased the proportion of apoptotic cells at early and advanced stages in a concentration-dependent manner (Fig. 2E-H). Next, we further found through Western blot analysis that CAR upregulated the expression levels of the pro-apoptosis-related proteins Bad, Bax, cleaved PARP, and cleaved caspase-3 in EC9706 cells and downregulated the expression level of the anti-apoptosis-related proteins Bcl-2, PARP, and caspase-3 (Fig. 2I-J). Caspase-3 is a key enzyme in cell apoptosis, and increased caspase-3 expression and activation are important elements in the apoptosis signal transduction pathway. To further study whether CAR is responsible for the pro-apoptotic effect of caspase-3 on oesophageal cancer cells, we used the general caspase inhibitor Z-VAD-FMK for experiments. The Western blot experiment revealed that the expression of related proteins was also reversed (Supplementary Fig. 1I-J). Next, we used flow cytometry apoptosis assays, and the results showed that the caspase-3 inhibitor group had a significantly decreased apoptotic rate of oesophageal cancer cells compared with the CAR treatment group (Supplementary Fig. 1K-L). In summary, these results indicate that CAR induced oesophageal cancer cell apoptosis through the mitochondria-related apoptotic pathway.

### CAR inhibited oesophageal cancer cell migration and invasion

**Figure 3:**
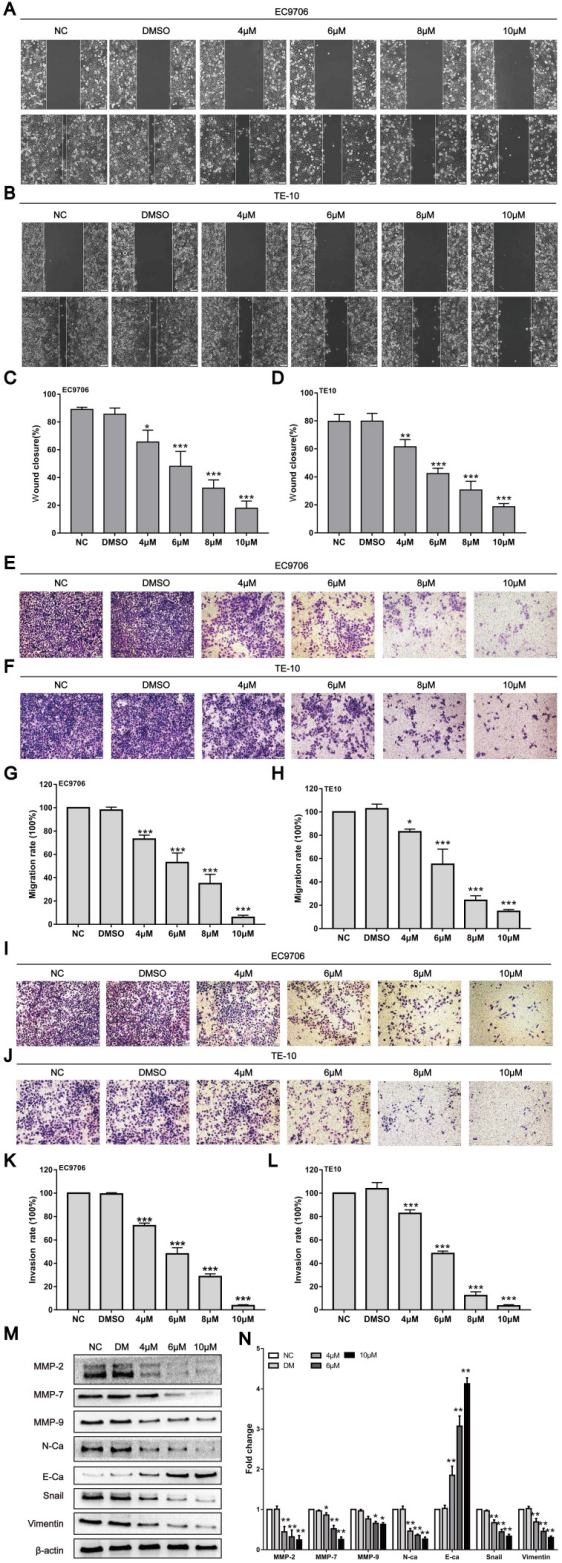
CAR inhibited oesophageal cancer cell migration and invasion. (A-D) A wound-healing assay showed the migratory abilities of EC9706 and TE10 cells treated with different concentrations of CAR or DMSO; the statistical results for cell migration width at 0 h and 24 h are shown. (E-H) A representative image of EC9706 and TE10 cells treated with different concentrations of CAR or DMSO in a transwell migration assay; migrating cells were counted under five randomly selected visual fields. (I-L) A representative image of EC9706 and TE10 cells treated with different concentrations of CAR or DMSO in a Transwell Matrigel invasion assay; invading cells were counted under five randomly selected visual fields. The above results proved that CAR inhibited the migration and invasion of oesophageal cancer cell lines EC9706 and TE10 in a concentration-dependent manner. (M and N) The expression levels of EMT-related marker proteins in EC9706 cells treated with different concentrations of CAR or DMSO were detected by Western blot with β-actin as the loading control.With increasing CAR concentrations, E-cadherin expression was upregulated, while the expression levels of vimentin, N-cadherin, EMT transcription factor Snail, MMP2, MMP7, and MMP9 were downregulated. All the above quantitative results are presented as the mean ± SD (n = 3, each group). *p < 0.05, **p < 0.01, ***p < 0.001 vs NC group.

Tumour progression mainly manifests as invasion and metastasis. However, whether CAR can affect the invasion and metastasis ability of oesophageal cancer cells is unclear. Subsequently, the effects of CAR on the invasion and metastasis ability of oesophageal cancer cells were investigated through wound healing and Transwell assays. The wound healing assay showed that within 24 h, the healing rate for EC9706 and TE10 oesophageal cancer cells gradually decreased with increases in the CAR concentration (Fig. 3A-D). Furthermore, the Transwell migration assay showed that compared with the control group, CAR treatment significantly inhibited oesophageal cancer cell migration (Fig. 3E-H). In addition, the Matrigel invasion assay indicated that CAR significantly inhibited the invasive ability of oesophageal cancer cells (Fig. 3I-L). These results all indicate that CAR significantly inhibited the migration and invasion of EC9706 cells and TE10 cells in a concentration-dependent manner. Epithelial-mesenchymal transition (EMT) is considered an important biological process for epithelial-derived malignant tumour cells to acquire the ability to migrate and invade 35. Therefore, we examined the expression of EMT-associated proteins by Western blot analysis. As shown in (Fig. 3M and N), in EC9706 cells, CAR upregulated the expression of E-cadherin and significantly downregulated the expression of N-cadherin and vimentin as well as the EMT transcription factor Snail. Matrix metalloproteinases (MMPs) are important proteolytic enzymes that can destroy histological barriers to tumour cell invasion and play key roles in tumour invasion and metastasis. Therefore, their role in tumour invasion and metastasis has received increasing attention. Western blot results confirmed that CAR significantly reduced the expression of MMP2, MMP7, and MMP9. In summary, CAR inhibited oesophageal cancer cell migration and invasion by inhibiting EMT and downregulating MMP expression.

### CAR inhibited the PI3K/AKT signalling pathway

**Figure 4:**
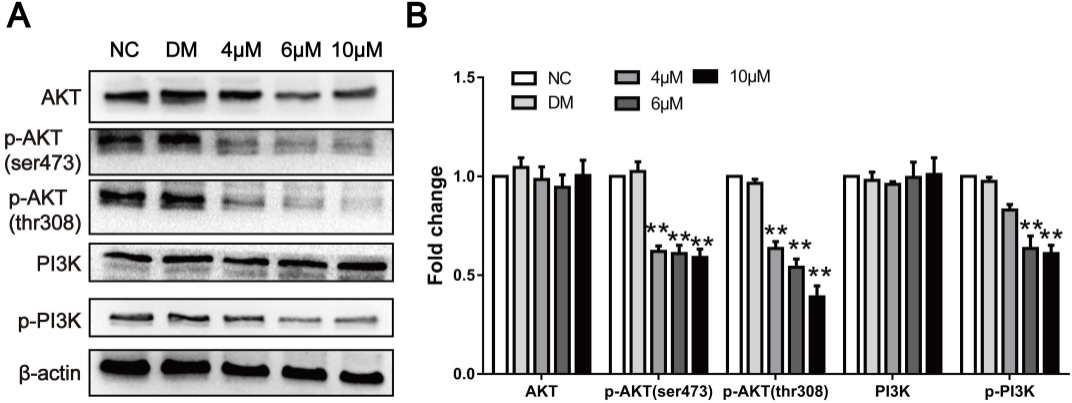
CAR inhibited the PI3K/AKT signalling pathway. (A and B) The protein levels of PI3K, p-PI3K and its downstream effector molecule AKT, and p-AKT were examined by Western blot in EC9706 cells treated with different concentrations of CAR or DMSO, and β-actin served as the loading control. With increasing CAR concentrations, the protein levels of p-PI3K and p-AKT decreased significantly, while the expression levels of PI3K and AKT remained unchanged. The data are presented as the mean ± SD (n = 3, each group). *p < 0.05, **p < 0.01 vs the NC group.

The proliferation and metastasis of tumour cells are the basis of cancer progression. To date, studies have shown that a large number of signalling pathways are involved in this process. Previous studies have shown that CAR exerts antitumour activities by targeting different pathways. To clarify the specific mechanism of action, we used Western blot analysis to verify whether CAR regulates the progression of oesophageal cancer through PI3K/AKT. Therefore, we examined the expression of pathway-associated proteins. As shown in (Fig. 4A and B), after treatment with CAR, the expression and phosphorylation levels of PI3K and the PI3K downstream effector molecule AKT in EC9706 cells significantly decreased in a concentration-dependent manner as expected, whereas the total protein level did not change significantly. These results indicate that CAR inhibited oesophageal cancer cell proliferation and migration by blocking the PI3K/AKT signalling pathway.

### 740Y-P reversed the inhibitory effect of CAR on oesophageal cancer cells

**Figure 5:**
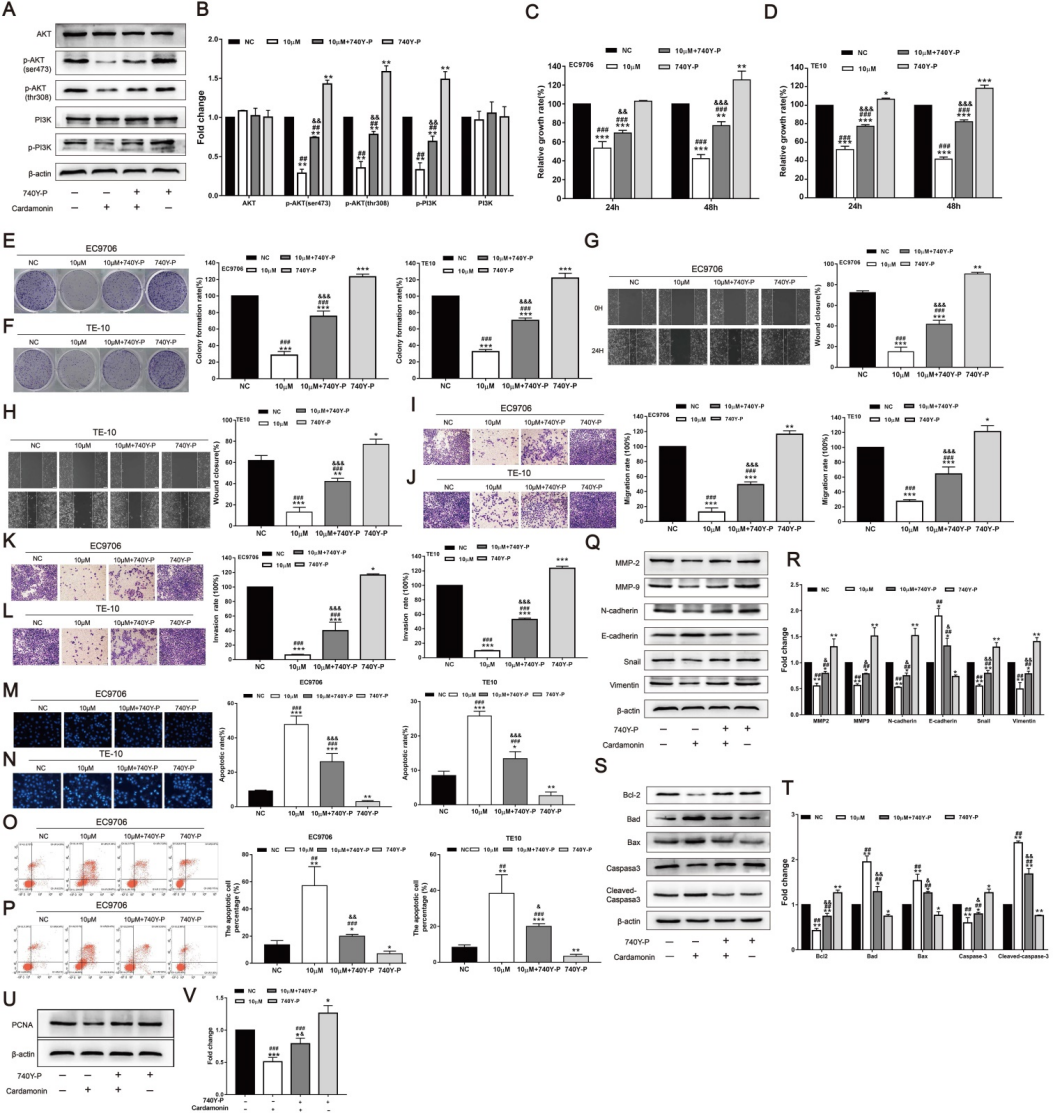
740Y-P reversed the inhibitory effect of CAR on oesophageal cancer cells. (A and B) The oesophageal cancer cell line EC9706 was incubated with CAR and the PI3K agonist 740Y-P or subjected to co-incubation, and Western blot results proved that 740Y-P could partially reverse the inhibitory effect of CAR on the PI3K/AKT pathway. Similarly, EC9706 and TE10 cells were treated with CAR and the PI3K agonist 740Y-P or with co-incubation. (C and D) MTT assay and (E and F) colony formation assay results confirmed that the PI3K agonist 740Y-P could partially reverse the antiproliferation effect of CAR. (G and H) Scratch healing assay, (I and J) Transwell migration assay, and (K and L) Transwell invasion assay results showed that the PI3K agonist 740Y-P can partially reverse the anti-migration and anti-invasion effects of CAR on oesophageal cancer cells. (M and N) Hoechst 33258 staining and (O and P) flow cytometry were used to verify the effect of CAR and 740Y-P or co-incubation on oesophageal cancer cell apoptosis, and the results showed that 740Y-P could partially reverse the apoptosis-promoting effect of CAR on EC9706 and TE10 cells. (Q-V) Western blot and quantitative analysis were used to detect the protein expression levels of Bcl2, Bax, Bad, caspase-3, cleaved caspase-3, MMP2, MMP9, N-cadherin, E-cadherin, Snail, vimentin, and PCNA after EC9706 cells were treated with 740Y-P and CAR or with co-incubation. The results showed that 740Y-P could partially reverse the expression of apoptosis-related proteins, EMT-related proteins, and PCNA. The data are expressed as the mean ± SD (n = 3, each group). Compared with the NC group, *p < 0.05, **p < 0.01, ***p < 0.001; Compared with the 740Y-P group, #p < 0.05, ## p < 0.01, ### p < 0.001; Compared with the Cardamonin group, & p < 0.05, && p < 0.01, &&& p < 0.001.

To further validate the function of the PI3K/AKT signalling pathway, the PI3K agonist 740Y-P was used, and the effects of CAR on the proliferation, invasion, migration, and apoptosis of oesophageal cancer cells were investigated. As shown in (Fig. 5A and B), after treatment with CAR and 740Y-P or co-incubation, 740Y-P eliminated the inhibitory effect of CAR on PI3K/AKT in oesophageal cancer cells. Next, we also performed MTT (Fig. 5C and D), colony formation (Fig. 5E and F), scratch healing (Fig. 5G and H), Transwell migration (Fig. 5I and J), Matrigel invasion (Fig. 5K and L), Hoechst 33258 staining (Fig. 5M and N), and flow cytometry apoptosis assays (Fig. 5O and P). After co-treatment with 740Y-P, the inhibitory effect of CAR on oesophageal cancer cells was significantly reversed. In addition, the expression of related proteins was also reversed (Fig. 5Q-V). In summary, these results further confirmed that CAR may inhibit oesophageal cancer cells through the PI3K/AKT signalling pathway.

### CAR inhibited the growth of EC9706 cells *in vivo*

**Figure 6:**
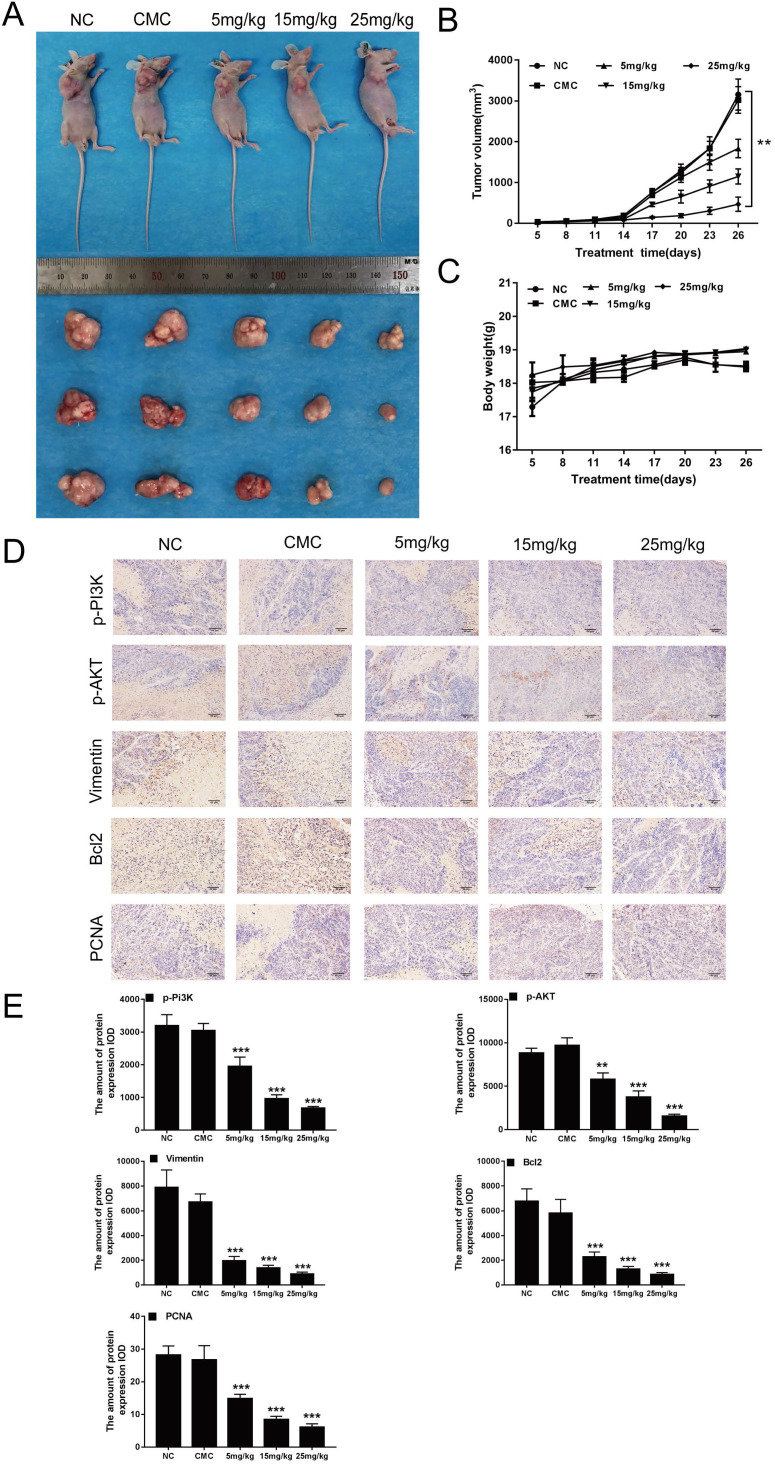
CAR inhibited the growth of EC9706 cells *in vivo*. (A and B) Nude mice were perfused with different concentrations of CAR, and tumour size was measured every three days. Compared with the control group, the CAR treatment group showed significantly inhibited tumour growth. (C) The weights of mice were measured every three days. Compared with that in the control group, weight in the CAR treatment group did not significantly change. (D and E) PCNA, p-PI3K, p-AKT, Bcl2, and vimentin were detected by immunohistochemistry, and with increasing CAR concentrations, the expression levels of PCNA, p-PI3K, p-AKT, Bcl2, and vimentin were downregulated. The quantitative data are presented as the mean ± SD (n = 3 in each group). *p < 0.05, **p < 0.01, ***p < 0.001 vs the NC group.

Previous studies have shown that CAR can inhibit the growth of oesophageal cancer cells *in vitro*. Thus, we investigated whether CAR has a similar effect *in vivo*. We used EC9706 cells to construct a mouse model of subcutaneous tumour formation. As shown in (Fig. 6A and B), with increasing doses of CAR, tumour growth was limited, but the weights of the mice did not show the same change (Fig. 6C). In addition, IHC staining of mouse tumour tissues showed that the protein expression of PCNA, Bcl2, vimentin, p-PI3K, and p-AKT was reduced (Fig. 6D and E), which was consistent with the results of the *in vitro* studies. To explore whether CAR has a toxic effect on mice, we analysed the important organs of mice in the CAR group. As shown in (Supplementary Fig. 2A), no difference in HE staining was observed between the treatment group and the control group. Similarly, we verified whether CAR can change blood biochemical indexes and blood routine indexes in mice. As shown in Table 1 and Table 2, no obvious difference was found between the blood routine indexes and blood biochemical indexes of mice in the treatment group and those of mice in the control group, demonstrating that CAR does not damage important organs or alter blood indexes. In summary, these data provide preliminary evidence for the therapeutic application of CAR in oesophageal cancer.

## Discussion

Oesophageal cancer is one of the most invasive malignancies. Although progress in surgical, chemotherapy, and radiation treatments has been achieved, the incidence of local invasion and distant metastasis is very high; notably, the five-year survival rate for patients with advanced oesophageal cancer is still below 20% 26. Due to their stable therapeutic effects and low toxic side effects, plant-derived compounds have received broad attention. For example, natural Chinese medicine compounds such as oridonin, sinomenine, apigenin, matrine, and sinomenine have been reported to have anti-oesophageal cancer effects through different mechanisms 5,27. As a natural traditional Chinese medicine compound, CAR can exert antitumour effects on a variety of tumour cells by targeting different signalling pathways. Previous studies have shown that traditional Chinese medicine herbs mainly exert antitumour effects by inhibiting tumour cell proliferation, migration, and invasion and by inducing apoptosis 27. This study also demonstrated that CAR inhibited the proliferation, migration, and invasion of oesophageal cancer cells and induced apoptosis. In addition, the inhibitory effect of CAR on oesophageal cancer cells was confirmed in subcutaneous tumourigenesis experiments in nude mice, and CAR played an antitumour role through the PI3K/AKT signalling pathway.

PCNA is an acidic nuclear protein involved in DNA synthesis and repair. It is considered a histological marker of cell proliferation and is overexpressed in various tumour tissues and cells. Thus, PCNA participates in a wide range of cellular processes, including DNA replication, DNA damage repair, mismatch repair, and cell cycle regulation. PCNA also plays important roles in apoptosis inhibition, chromatin metabolism, and gene expression 28-30. In this study, we found that CAR showed an antiproliferative effect on EC9706 and TE10 cells in time- and dose-dependent manners. Western blot analysis further confirmed that CAR significantly downregulated the expression of PCNA in EC9706 cells.

The effects of traditional Chinese medicine herbs on tumour cells include not only inhibition of cell proliferation but also promotion of apoptosis. Apoptosis, or programmed cell death, is a process in which cells stop growth and division and is finely regulated at the gene level; cell apoptosis mainly includes an intrinsic pathway (mitochondrial pathway) and an extrinsic pathway (death receptor pathway) 31-32. The intrinsic apoptotic pathway is mediated by intracellular signals. When the intrinsic pathway (mitochondria) is triggered, the pro-apoptotic proteins Bax and Bad in the Bcl2 family are activated, and the anti-apoptotic proteins Bcl2 and Bcl-xL are downregulated; thus, the Bax to Bcl2 ratio becomes imbalanced. The imbalanced Bax/Bcl2 ratio may lead to increased mitochondrial membrane permeability and the release of cytochrome C. Released cytochrome C binds to cytoplasmic apoptotic protease-activating factor-1 (Apaf-1), forming an apoptotic complex. This complex further recruits the apoptosis promoter, pre-caspase-9, into the caspase recruitment domain, where self-activation and self-proteolysis occur, thus activating downstream caspase-3 and caspase-7; activated caspase-3 can further lyse PARP, eventually leading to apoptosis 33-34. In this study, we treated EC9706 and TE10 cells with different concentrations of CAR. The results from the flow cytometry and Hoechst staining experiments showed that CAR significantly promoted apoptosis. Moreover, the anti-apoptosis-related protein Bcl2 decreased in a dose-dependent manner, while the pro-apoptosis-related proteins Bad, Bax, cleaved caspase-3, and cleaved PARP increased in a dose-dependent manner. In addition, to further verify that CAR promotes the apoptosis of oesophageal cancer cells through mitochondrial apoptosis, Z-VAD-FMK, a general caspase inhibitor, was used to treat oesophageal cancer cells in advance, and the results proved that Z-VAD-FMK could reverse the apoptosis induced by CAR. Therefore, the results showed that the induction of mitochondrial apoptosis was a manifestation of the anticancer activity of CAR.

Metastasis, which is the spread of cancer cells from the primary tumour to other organs, is the most common biological process of tumour progression, and EMT is one of the important mechanisms. EMT is regarded as a process in which tumour cells transform from a static epithelial phenotype to a migratory mesenchymal phenotype. EMT involves a variety of tumour functions, including tumour initiation, malignant progression, and tumour cell migration and metastasis 35-36. EMT biomarkers include mesenchymal markers such as N-cadherin, vimentin, Slug, and Snail as well as epithelial markers such as E-cadherin and catenin. E-cadherin is a member of the classical cadherin family. E-cadherin regulates cell-cell adhesion and prevents cell migration through physical functions. Studies have shown that loss of the expression of E-cadherin, as a classical tumour suppressor, is closely related to tumour progression and metastasis 37. E-cadherin deficiency is often accompanied by upregulation of N-cadherin expression. As a calcium-dependent adhesion molecule, N-cadherin is resistant to protease hydrolysis and promotes tumour cell metastasis in the presence of calcium ions. N-cadherin expression promotes *in situ* shedding of tumour cells and overcomes the antitumour effect of E-cadherin 38. Snail is closely associated with tumour metastasis. High Snail expression enhances cell invasiveness by upregulating mesenchymal markers and downregulating epithelial markers, mainly by recruiting specific chromatin modifications to the E-cadherin promoter, thereby silencing E-cadherin expression and inducing EMT 39. Vimentin is an important component of the intermediate filament protein family and is commonly expressed in normal mesenchymal cells. Vimentin is considered a marker of EMT 40. MMPs are calcium-dependent zinc-containing endopeptidases and are key proteolytic enzymes that degrade the extracellular matrix and basement membrane, allowing cancer cells to penetrate and infiltrate the substroma, thus promoting tumour progression and invasion 41. MMP2, MMP7, and MMP9 are members of the MMP family and are mainly involved in tumour invasion and metastasis. *In situ* gelatine gel profiles show that MMP2 and MMP9 gelatinolytic activity is enhanced in oesophageal squamous cell carcinoma and closely associated with vascular invasion in oesophageal cancer 42. Through wound healing and Transwell assays, we demonstrated the inhibitory effects of CAR on oesophageal cancer cell invasion and migration. Western blot analysis showed that CAR significantly downregulated Snail, vimentin, N-cadherin, MMP2, MMP7, and MMP9 expression and upregulated E-cadherin expression. Therefore, this study confirmed that the inhibitory effects of CAR on oesophageal cancer cell invasion and migration might be related to EMT for the first time.

PI3K, a family of unique intracellular lipid kinases, can be divided into three classes, the most extensively studied of which is class I PI3K. Class I PI3K is a heterodimer composed of a catalytic subunit (p110) and regulatory subunits (p85). The regulatory subunits contain SH2 and SH3 structure domains. Four catalytic subunit subtypes exist: p110α, p110β, p110γ, and p110δ 43. When the PI3K signalling pathway is activated, receptors activate the SH2 structure domain of the regulatory subunit (p85) and recruit the catalytic subunit (p110), thus catalysing the conversion of phosphatidylinositol 4,5-diphosphate (PIP2) on the inner surface of the membrane into phosphatidylinositol 3,4,5-triphosphate (PIP3). As a second messenger, PIP3 can bind to the signal proteins AKT, phosphatidylinositol-dependent protein kinase 1 (PDK1), and phosphatidylinositol-dependent protein kinase 2 (PDK2). PDK1 and PDK2 contain intracellular pleckstrin homology (PH) domains that promote the phosphorylation of threonine 308 and serine 473 of AKT, respectively, leading to AKT activation 43-45. AKT, also known as protein kinase B, is a key regulator of cell growth and can regulate anti-apoptotic proteins and cell proliferation, increase Bad phosphorylation, and inactivate Bad, thereby inhibiting apoptosis 46. In recent years, many studies have shown that the PI3K/AKT signalling pathway is abnormally activated in a variety of malignant tumours and that abnormal activation of this pathway is clearly associated with tumourigenesis, cancer progression, and drug resistance 24. Therefore, the PI3K/AKT pathway is considered a promising anticancer target. To date, the US FDA has approved PI3K-targeting drugs such as idelalisib, copanlisib, and duvelisib for the treatment of relapsed or refractory chronic lymphocytic leukaemia (CLL) 47. Traditional Chinese medicine herbs have been reported to be able to inhibit the PI3K/AKT pathway and thus inhibit oesophageal cancer cell proliferation and promote apoptosis; for example, Liu et al. showed that ginsenoside Rk3 mediates apoptosis and autophagy by regulating the PI3K/AKT pathway and has anti-oesophageal cancer effects *in vivo* and *in vitro* 48. Zhou et al. showed that through inhibition of the PI3K/AKT pathway, CAR causes G2/M phase cell cycle arrest and loss of invasion and migration phenotypes in non-small cell lung cancer cells 19. The above studies demonstrate that as a traditional Chinese medicine herb, CAR can inhibit the growth of cancer cells by blocking PI3K/AKT. However, no studies have demonstrated the role of CAR in oesophageal cancer cells. To better understand the mechanism underlying the inhibition of oesophageal cancer cell growth and the promotion of apoptosis by CAR, we investigated the effects of CAR on the PI3K/AKT pathway. Our results showed that after CAR treatment, the protein expression of PI3K and AKT was downregulated in a dose-dependent manner; when oesophageal cancer cells were co-treated with the PI3K agonist 740Y-P and CAR, the inhibition of cell proliferation, invasion, migration, and EMT and the promotion of cell apoptosis by CAR were reversed.

**Figure 7:**
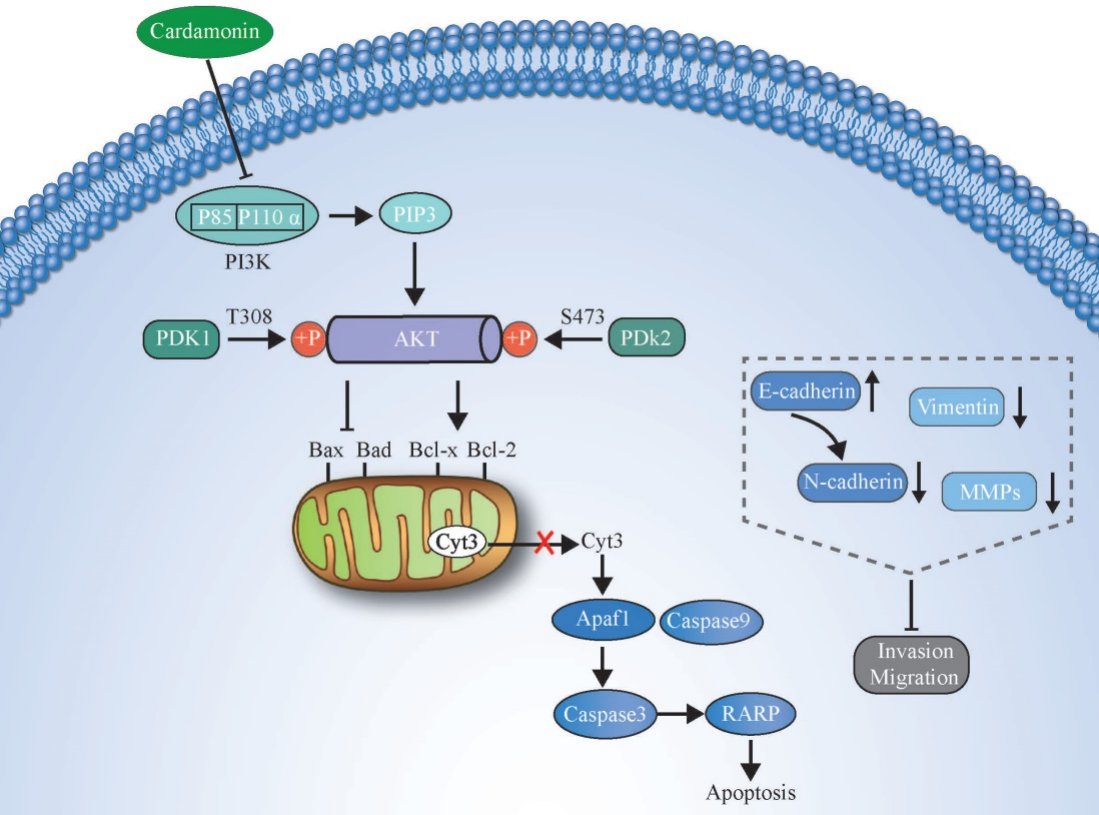
An illustration showing how CAR exerts anticancer activity in oesophageal cancer cells by suppressing the PI3K/AKT signalling pathway.

In summary, we demonstrated that CAR has strong effects on inhibiting the proliferation and promoting the apoptosis of human oesophageal cancer cells. In addition, through reversal of the EMT process, CAR causes the loss of invasion and migration phenotypes of oesophageal cancer cells. The possible mechanism may be that CAR exerts its anticancer effect by inhibiting the PI3K/AKT pathway. The potential molecular mechanism is shown in (Fig. 7). Our data indicate that CAR can be a natural option for oesophageal cancer treatment and suggest that targeting the PI3K/AKT pathway may be a promising cancer treatment strategy.

## Supplementary Material

Supplementary figures.

## Figures and Tables

**Table 1:** Level of blood routine related indexes in experimental nude mice

	NC	CMC	5 mg/kg	15 mg/kg	25 mg/kg
WBC (×10^9^/L)	3.83±0.13	3.93±0.12	3.90±0.17	3.82±0.07	3.80±0.14
LYM (%)	70.27±4.02	65.67±2.15	70.80±4.07	72.30±3.15	74.13±1.53
Mon (%)	3.10±1.47	4.17±0.90	3.77±1.95	3.17±1.01	3.20±0.87
Gran (%)	26.63±5.20	28.83±0.45	25.43±2.58	24.53±2.28	23.53±2.25
RBC (×10^12^/L)	6.60±0.38	7.91±1.53	6.91±1.49	6.13±0.08	6.42±0.19
HGB (g/L)	145.00±3.00	127.67±4.04	131.33±6.51	137.00±19.00	142.33±5.69
HCT (%)	40.17±1.81	45.67±9.79	39.87±7.80	35.77±1.86	34.30±2.17
MCV (fL)	60.90±3.12	57.53±1.56	57.63±1.50	58.33±2.78	56.03±1.56
MCH (pg)	21.43±2.22	21.37±1.22	20.07±0.93	19.40±2.07	17.93±0.49
MCHC (g/L)	353.00±19.08	371.00±11.27	348.00±12.77	332.33±19.09	336.00±7.00
PLT (×10^9^/L)	1099.00±63.15	1020.00±73.90	1009±44.24	1074.00±42.80	1077.00±42.58

Note: WBC: White blood cell, LYM: Lymphocyte, Mon: Monocyte, Gran: Granulocyte, RBC: Red blood cell, HGB: Hemoglobin, HCT: Hematocrit, MCV: Mean corpuscular volume, MCH: Mean corpuscular hemoglobin, MCHC: Mean corpuscular hemoglobin concentration, PLT: Platelet. Results are expressed as Mean±SD (n=3).

**Table 2:** Serum ALT, AST and CR expression levels in experimental nude mice

	NC	CMC	5 mg/kg	15 mg/kg	25 mg/kg
ALT (U/L)	22.71±1.16	23.64±1.87	25.97±1.19	23.50±2.88	21.04±2.52
AST (U/L)	59.03±3.36	61.60±2.31	57.56±2.19	55.38±3.26	57.20±4.16
CR (μmol/L)	56.00±3.87	55.24±8.92	51.41±4.33	49.61±6.54	53.34±7.72

Note: ALT: Alanine aminotransferase, AST: Aspartate aminotransferase, CR: Creatinine. Results are expressed as mean±SD (n=3).
