# Supplementary material for: Cardamonin inhibits the progression of oesophageal cancer by inhibiting the PI3K/AKT signalling pathway
Source: J Cancer. 2021 Apr 24;12(12):3597–610. doi: 10.7150/jca.55519 (PMC8120183; doi:10.7150/jca.55519)
Supplement: Supplementary file 1 — Supplementary figures. [file jcav12p3597s1.pdf]

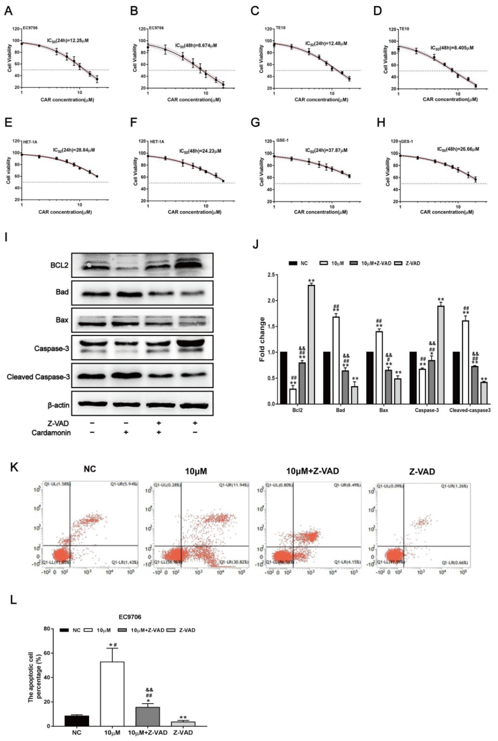

Supplementary Figure 1 (A-D) EC9706 and TE10 cells were treated with different concentrations of CAR or DMSO, and cell viability and the IC50 at 24 h and 48 h were examined by MTT assay. (E-H) HET-1A and GES-1 cells were treated with different concentrations of CAR or DMSO, and cell viability and the IC50 at 24 h and 48 h were examined by MTT assay. (I-J) EC9706 cells were incubated with CAR or the caspase inhibitor Z-VAD-FMK or co-incubated, and mitochondrial apoptosis pathway-related proteins were detected by Western blot. The results showed that Z-VAD-FMK could partially reverse the expression of Bcl2, Bax, Bad, caspase-3, and cleaved caspase-3. (K and L) The apoptosis of EC9706 cells after applying the caspase inhibitor Z-VAD-FMK was detected by flow cytometry, and the results showed that Z-VAD-FMK could partially reverse the pro-apoptosis effect of CAR.

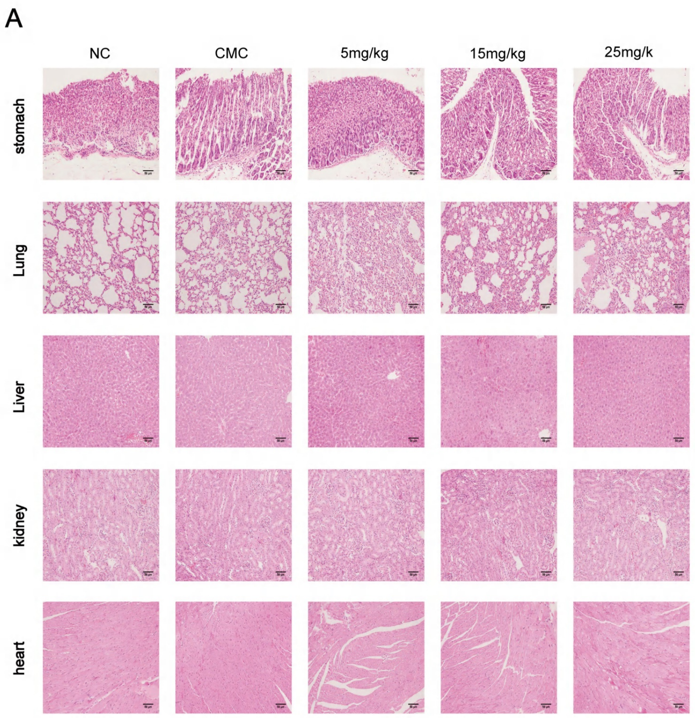

Supplementary Figure 2 (A) Mice that developed tumours were treated with CAR, and stomach tissue, liver tissue, lung tissue, kidney tissue, and heart tissue were subjected to haematoxylin-eosin (HE) staining. The results showed no difference in HE staining between the treatment group and the control group.
